# Effect of smoking cessation on tooth loss: a systematic review with meta-analysis

**DOI:** 10.1186/s12903-019-0930-2

**Published:** 2019-11-12

**Authors:** Maria Luisa Silveira Souto, Emanuel Silva Rovai, Cristina Cunha Villar, Mariana Minatel Braga, Cláudio Mendes Pannuti

**Affiliations:** 1 Division of Periodontics, University of São Paulo, School of Dentistry, Av. Prof. Lineu Prestes, 2227, São Paulo, SP 05508-000 Brazil; 2 Department of Dentistry, Periodontics Research Division, University of Taubaté, Taubaté, São Paulo, Brazil; 3 Division of Pediatric Dentistry, University of São Paulo, School of Dentistry, São Paulo, Brazil

**Keywords:** Tobacco, Cigarette smoking, Tobacco use cessation, smoking cessation, Tooth loss, periodontitis, Meta-analysis

## Abstract

**Background:**

Smoking is a major risk factor for periodontitis and tooth loss. Smoking cessation has a positive impact in periodontal treatment. However, so far, no systematic review has evaluated the effect of smoking cessation on tooth loss. Therefore, this review aimed to evaluate if smoking cessation reduces the risk of tooth loss.

**Methods:**

Observational (cross-sectional and longitudinal) studies that investigated the association between smoking cessation and tooth loss were included. MEDLINE, EMBASE and LILACS databases were searched for articles published up to November 2018. Pooled results for subgroups of current and former smokers were compared in meta-analysis. Meta-regression was used to test the influence of smoking status on estimates and explore the heterogeneity.

**Results:**

Of 230 potentially relevant publications, 21 studies were included in the qualitative review and 12 in the quantitative analysis. Meta-analysis of cross-sectional studies did not show any differences between former and current smokers in the chance of losing 1 or more teeth (OR = 1.00; 95% CI = 0.80 to 1.24, I^2^ = 80%), losing more than 8 teeth (OR = 1.02; 95% CI = 0.78 to 1.32, I^2^ = 0%) or being edentulous (OR = 1.37; 95% CI = 0.94 to 1.99, I^2^ = 98%). Meta-analysis from longitudinal studies showed that, when compared to never smokers, former smokers presented no increased risk of tooth loss (RR = 1.15; 95% CI = 0.98 to 1.35, I^2^ = 76%), while current smokers presented an increased risk of tooth loss (RR = 2.60; 95% CI = 2.29 to 2.96, I^2^ = 61%). Meta-regression showed that, among former smokers, the time of cessation was the variable that better explained heterogeneity (approximately 60%).

**Conclusions:**

Risk for tooth loss in former smokers is comparable to that of never smokers. Moreover, former smokers have a reduced risk of tooth loss, when compared to current smokers.

## Background

Cumulative evidence from cross-sectional and cohort studies supports a causal relationship between cigarette smoking and the initiation and progression of periodontitis [1–5]. Smokers present greater extent and severity of periodontitis [6, 7]. Conversely, smoking cessation has a positive impact in the outcomes of non-surgical periodontal therapy [8–12].

Tooth loss is the final outcome of periodontal disease. It is associated with loss of masticatory function [13], lack of self-esteem and impaired social interactions due to limited aesthetic appearance [14, 15]. Consequently, tooth loss has a negative impact on oral health-related quality of life [16]. Smokers are more likely to lose their teeth than non-smokers [17, 18], as a result of their increased severity of periodontitis. Cross-sectional [19, 20] and prospective studies [21, 22] have also suggested that former smokers have a significantly lower risk of tooth loss than current smokers.

Previous reviews have addressed the association between smoking and tooth loss [17, 18]. However, so far, no review has focused on the effect of smoking cessation on tooth loss. Further, there is no meta-estimate to quantify the impact of smoking cessation on tooth loss. Therefore, the aim of this systematic review was to answer the following focused question: “Does smoking cessation reduce the risk of tooth loss in former smokers, when compared to current smokers?”

## Methods

This review has been prepared according to the Preferred Reporting Items for Systematic Reviews and Meta-Analyses (PRISMA) guidelines [23] and Meta-analysis of Observational Studies in Epidemiology (MOOSE) guidelines [24]. The protocol was registered in the International Prospective Register of Systematic Reviews – PROSPERO (CRD42018085095).

### Eligibility criteria

Only observational studies (cross-sectional and longitudinal studies) were included in this systematic review because, for ethical reasons, there are no randomized clinical trials with a control group that did not receive smoking cessation therapy. The inclusion criteria were as follows: a) original studies published in English; b) data comparing former smokers with current smokers and never smokers; c) studies that had tooth loss as an outcome.

We excluded narrative reviews, case series, case reports, in vitro and animal studies. Further, we excluded studies that did not include former smokers in the analysis, or that combined former smokers with never smokers or current smokers, or that did not associate smoking with tooth loss (e.g., smoking was used only for adjustment).

### Search strategy

An electronic literature search was conducted in the following databases: MEDLINE (PubMed), Web of Science and Cochrane Library in September 2019. The following search strategy was used: ((((((((((epidemiology) OR observational study) OR longitudinal) OR cohort) OR cross-sectional) OR prospective) OR retrospective)) AND (((((((tooth) OR tooth [MeSH Terms]) OR tooth loss) OR tooth survival) OR periodontitis) OR periodontal disease) OR Periodontal Diseases [MeSH Terms])) AND ((((((((tobacco) OR Tobacco Use Disorder [MeSH Terms]) OR cigarette smoking) OR tobacco products) OR smoking cessation) OR smoking [MeSH Terms]) OR smoking cessation [MeSH Terms]) OR tobacco use cessation)). We also conducted a hand search of references lists from included publications.

In the first phase, two reviewers (MLSS and ESR) screened independently titles and abstracts identified by the search strategy. Disagreements were resolved by discussion or, if necessary, by the decision of a third reviewer (CMP). In the second phase, the same reviewers screened full texts of the studies that met inclusion criteria, or those with unclear information in the title and abstract. Reasons for rejection of studies were recorded for each report.

### Data extraction

The following items were extracted from the publications that met inclusion criteria: author, year, country, study design, sample size, measures of exposure (smoking status), measures of outcome (tooth loss), results, conclusions, conflict of interest and source of funding. Authors of the included studies were contacted for missing, relevant data.

### Risk of bias

Risk of bias of cohort studies was assessed using a modified version of the Newcastle-Ottawa scale (NOS) [25]. For cross-sectional studies, we adapted the Modesti et al. (2016) version of the NOS scale [26].

The NOS for cohort studies comprised 10 questions about selection of the study groups (i.e. representativeness of current and former smokers), comparability of the groups, outcome (criteria used to assess tooth loss and adequacy of follow-up) and statistical analysis. The scores ranged from 0 to 11. Studies with 9–11 stars were arbitrarily rated as low risk of bias, 6–8 stars moderate risk of bias and < 6 high risk of bias.

The NOS for cross-sectional studies comprised 07 questions about selection of the study groups (i.e. representativeness of the sample), comparability of the groups, outcome (criteria used to assess tooth loss) and statistical analysis. The scores ranged from 0 to 10. Studies with 7–10 stars were arbitrarily rated as low risk of bias, 5–6 stars moderate risk of bias and < 5 high risk of bias.

### Summary measures and synthesis of results

Analyses of data extracted from cross-sectional studies were carried out using software Review Manager (RevMan) (Version 5.3. Copenhagen: The Nordic Cochrane Centre, The Cochrane Collaboration, 2014). Random-effects meta-analyses were conducted for the following outcomes: loss of one or more teeth, loss of more than eight teeth and being edentulous. The estimates were presented as pooled odds radios (ORs) and their respective 95% confidence intervals (CIs). Heterogeneity was tested using the Cochran’s Q test and quantified using the I-square test (level of inconsistency) and Tau^2^ (estimate of between-study variance).

Meta-analyses were performed to assess the risk of tooth loss among subgroups of former and current smokers, compared to the control group (never smokers). OR values for studied groups (former and current smokers) were converted into LogOR and results from individual studies were pooled using a random-effects model. Former and current smokers were considered as different subgroups and contrasted with never smokers. Differences between subgroups (subsets) were also tested based on random-effects models. Meta-analysis used the inverse variance method and the DerSimonian-Laird estimator for Tau^2^. The pooled results were estimated using the Risk Ratio (RR), Relative Risk and 95% CIs. Heterogeneity was tested similarly to the cross-sectional studies.

Meta-regressions were used to test the influence of different moderators (age, time of cessation and dropout rates) on pooled estimates. After testing each variable in the model, residual heterogeneity (I^2^) and amount of heterogeneity accounted for each variable (R^2^) could be calculated. Funnel plot visual analysis and linear regression test of funnel plot asymmetry were used to assess publication bias of the longitudinal studies. Both meta-regressions and publication bias investigation were performed considering subgroups separately.

Data analyses of longitudinal studies were performed using the “meta” and “metafor” packages, R software (R Studio, Version 1.0.143).

## Results

### Search results and excluded trials

**Fig. 1:**
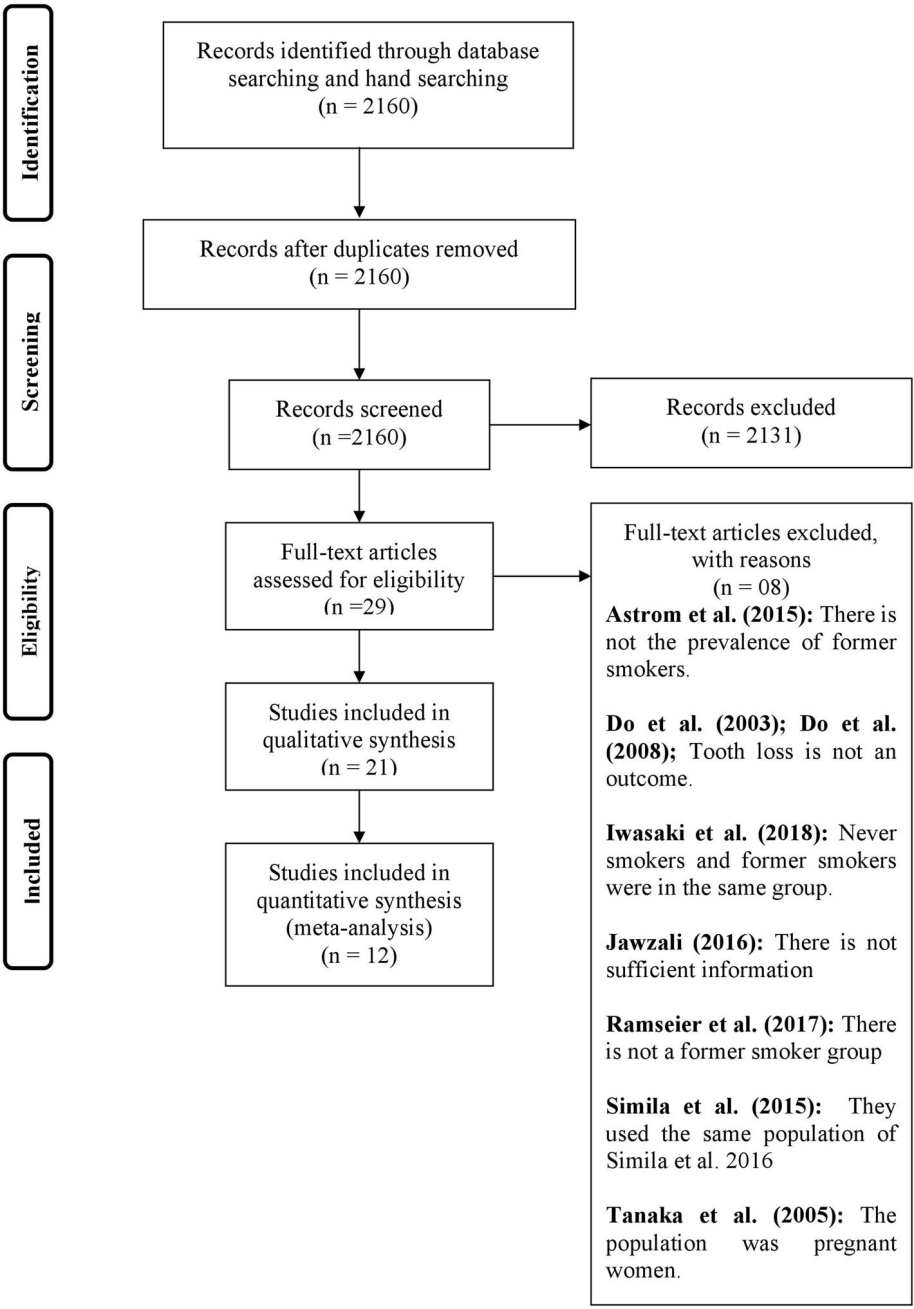
Flow-chart of studies screened, retrieved, included and analyzed in the systematic review and subsequent meta-analyses

From a total of 2160 papers identified from electronic databases and hand searching, 2131 were excluded after review of titles or abstract. In the second phase, 29 papers [3, 5, 19–22, 27–49] were read in full. After evaluation of the full report, eight [5, 30, 44–49] were excluded. At the end, 21 publications [3, 19–22, 27–29, 31–43] were included in this review. Of these, 12 [20, 27, 28, 31, 33–35, 38, 40–43] presented data that could be analyzed in meta-analysis (Flowchart- Fig. 1). Only observational studies (cross-sectional and longitudinal studies) were included because there was no randomized clinical study with tooth loss as outcome.

### Included studies

#### Cross-sectional studies

Fourteen cross-sectional studies were included in this review [19, 20, 27–29, 31–39]. Their characteristics are depicted in Table 1. A total of 567,491 individuals from both sexes, ranging 18–99 years were included.

**Table 1:** Characteristics of the cross-sectional studies included in this review (*n* = 14 studies)

Author (Country)	Subjects characteristics	Smoking status assessment	Outcome assessment	Main findings
Albandar et al. 2010 (EUA)	705 patients (age range of 21 to 91 years; mean 56.9 ± 16.4 years) from the Baltimore Longitudinal Study	Structured interview *Infrequent / non-smokers*: quit smoking cigarettes after smoking less than 10 years / no history of smoking *Current cigarettes smokers*: smoke daily *Former heavy smokers*: smoked cigarettes daily for 10+ years and quit	Clinical examination. Outcome: Number of missing teeth	Mean (S.E.) number of missing teeth: *Non-smokers* (*n* = 475): 2.8 (0.28) *Current smokers* (*n* = 36): 5.1 (0.78) *Former heavy smokers* (*n* = 167): 3.9 (0.43)
Arora et al. 2010 (Australia)	99,663 (45 years and older) participants from the 45 and Older Study who responded the question about tooth loss	Self-reported questionnaire *Never smokers*: not defined by the authors *Current smokers*: heavy smokers (> 20 cigarettes *per* day) and those who smoke < 20 cigarettes *per* day. *Former smokers*: subjects were divided in categories of years since smoking cessation (< 10, 10–19, 20–29, 30 or more)	Self-reported questionnaire. Outcome: Number (%) of edentulous subjects	Number (%) of edentulous subjects *Never smokers* (*n* = 56,203): 4898 (8.7%) *Current smokers* (*n* = 7230): 939 (13%) *Former smokers* (*n* = 32,368): 3706 (11,4%)
Cunningham et al. 2016 (EUA)	439,637 respondents (18 to 98 years) from the 2021 Behavioral Risk Factor Surveillance System	Telephone interview *Never smokers*: smoked < 100 cigarettes during their lifetime *Current cigarette smokers*: smoked > 100 cigarettes during their life-time and reported smoking “every day” or “some days” at the time of the interview. *Former smokes*: reported ever smoking > 100 cigarettes but reported smoking “not at all” at the interview.	Telephone interview Question: “How many of your permanent teeth have been removed because of tooth decay or gum disease?” Outcome: number of teeth lost (in categories: none; 1 to 5; > 6 but not all; all)	Number (%) of edentulous subjects (all teeth lost) *Never smokers* (*n* = 239,920): 67,418 (28.1%) *Current smokers* (*n* = 72,210): 23,107 (32.0%) *Former smokers* (*n* = 127,507): 50,875 (39.9%)
Hanioka et al. 2007 (Japan)	3999 (> 40 years) from Survey of Dental Disease (SDD) and National Nutrition Survey (NSS)	Dietitian-applied questionnaire *Non-smoker*: never smoked or smoked no more than 100 cigarettes *Current smoker*: smokes currently and has smoked more than 100 cigarettes *Former smoker*: has previously smoked more than 100, but does not currently smoke	Clinical examination Outcome: number of subjects with less than 19 teeth	Number (%) of subjects having less than 19 teeth *Non-smoker* (*n* = 2502): 928 (37.1%) *Current smoker* (*n* = 922): 344 (37.3%) *Former smoker (n* = 575): 219 (38.1%)
Mai et al. 2013 (EUA)	1106 postmenopausal (mean 66.9 ± 7.1 years) women enrolled in the Buffalo Osteoporosis and Oral Bone Loss (OsteoPerio) Study	Self-administered questionnaire *Never smokers:* not defined by the authors *Current smokers:* light smokers (first tertile,< eight pack-years); Moderate smokers (second tertile, eight to 25 pack-years); Heavy smokers (third tertile, ≥ 26 pack-years). *Former smokers:* not defined by the authors	Clinical examination Outcome: number of subjects with any tooth loss	Number (%) of subjects with any tooth loss *Never smokers (n* = 593): 476 (80,2%) *Current smokers (n* = 40): 32 (80%) *Former smokers (n* = 473): 392 (82,8%)
Mundt et al. 2007 (Germany)	4310 individuals (20 to 79 years) from the Study of Health in Pomerania (SHIP-0)	Interview *Always nonsmoker:* not defined by the authors *Current smoker*: maximum quantity of cigarettes smoked per day over a year was classified as < 10 cigarettes/ day, 10 to 19 cigarettes/day, and > 20 cigarettes/day. *Former smoker:* not defined by the authors	Clinical examination Outcome: few teeth in relation to their age. The 15% of participants with the highest number of missing teeth in each 5-year age group were considered as cases.	Percentage of the case group (*n* = 355): *Never smokers: 19.7%* *Current smokers (n* = 196): < 10: 11.3% 10–19: 22.3% > 20: 21.7% *Former smokers (n* = 89): < 10 (*n* = 34): 9.6% 10–19 (*n* = 20): 5.6% > 20 (n = 35): 9.9%
Mussachio et al. 2007 (Italy)	3054 subjects > 65 years (mean age: 76.8 ± 8.7) from Porgetoo Veneto Anziani (Pro.V.A.) Study	Home interview *Never smoker:* not defined by the authors *Current smoker:* not defined by the authors *Former smoker:* not defined by the authors Former and current were subdivided by number of cigarettes/day in light, mild, and heavy smokers (< 10; 10–20; > 20 cigarettes/day)	Clinical examination Outcome: Prevalence of edentulism and number of remaining teeth, applied as dichotomous (none versus at least 1) or categorical (0; 1–7; 8–19; > 20).	Number (%) of edentulous subjects *Never (n* = 1900): 1144 (60.2%) *Current (n* = 260): 24 (9.4%) *Former (n* = 895): 273 (30.5%)
Ojima et al. 2007 (Japan)	1314 (20 to 39 years) subjects from the Nation Nutrition Survey (NNS) and the Survey of Dental Diseases (SDD)	Questionnaire (interview) *Nonsmoker*: experimental smoker or has never smoked cigarettes *Current smoker*: currently smokes cigarettes daily or occasionally *Former smoker*: has smoked cigarettes at some point in life, but currently does not smoke.	Clinical examination Outcome: Prevalence of participants with1+ tooth loss	Number (%) of subjects with 1+ tooth loss Overall *Nonsmoker (n* = 847): 236 (27.9%) *Current smoker (n* = 389): 158 (40.6%) *Former smoker (n* = 78): 18 (23.1%)
Randolph et al. 2001 (EUA)	3050 noninstitutionalized Mexican Americans age 65 to 99 from the Hispanic Established Population for the Epidemioligical Study of the Eldery survey.	Interview *Nonsmokers:* never smoked or reported having smoked fewer than 100 cigarettes in their lifetime *Current smokers:* currently smoking *Former smokers:* had smoked more than 100 cigarettes in their lifetime but were not currently smoking	Questionnaire (self-reported) Question “How many of your own teeth do you still have?” Categories: all, about three-quarters, about half, about one-quarter, none Outcome: Prevalence of tooth loss.	Number (%) of edentulous subjects (*0 teeth*) *Nonsmoker (n* = 1707): 397 (23,2%) *Current smoker (n* = 369): 131 (35,5%%) *Former smoker (n* = 826): 267 (32,3%) Number (%) of subjects with tooth loss (1+) *Nonsmokers (n = 1707)*: 1311 (76,8%) *Current (n = 369)*: 240 (65%) *Former (n* = 868): 598 (68,9%)
Simila et al. 2016 (Filand)	5540 subjects from Northern Finland Birth Cohort Study 1996. The study used data from the 46-year follow-up (carried out in 2012–2014).	Mailed questionnaire *Never smokers*: had smoked daily for less than one year in their lifetime and were not smokers at the time of the follow-up *Current smokers*: reported smoking at least occasionally. *Former smokers*: had smoked daily for at least one year, but had quit smoking and were not smokers at the time of the study	Self-reported Questionnaire Number (%) of subjects in the following categories of number of teeth: 0–27 or 28–32	*Number (%) of subjects in each category of number of teeth:* *Never smoker* (*n* = 3062) 0–27: 933 (35%) 28–32: 1763 (65%) *Current smoker (n* = 1757) 0–27: 742 (49%) 28–32: 758 (51%) *Former smoker* (*n* = 1525) 0–27: 559 (42%) 28–32: 785 (58%)
Torrungruang et al. 2012 (Thailand)	1463 individuals (50–73 years old) from the cross-sectional data of senior employess and retired personnel of the Electricity Generating Authority of Thailand.	Self-reported questionnaire *Non-smokers*: had never smoked or had smoked fewer than 100 cigarettes in their lifetime. *Current smokers*: currently smoked at the time of examination (smoked at least 100 cigarettes in their lifetime) *Former smokers*: had quit smoking prior to the time of examination (smoked at least 100 cigarettes in their lifetime)	Clinical examination Outcome: number of remaining teeth	*Number of remaining teeth:* *Non-smoker (n* = 477) Maxillary anterior teeth: 2.8 ± 0.03 Maxillary posterior teeth: 3.2 ± 0.05 Mandibular anterior teeth: 2.9 ± 0.02 Mandibular posterior teeth: 2.9 ± 0.05 *Current smoker (n* = 272) Maxillary anterior teeth: 2.6 ± 0.05 Maxillary posterior teeth: 2.9 ± 0.07 Mandibular anterior teeth: 2.8 ± 0.03 Mandibular posterior teeth: 2.8 ± 0.07 *Former smoker (n* = 714) Maxillary anterior teeth: 2.7 ± 0.03 Maxillary posterior teeth: 2.9 ± 0.04 Mandibular anterior teeth: 2.9 ± 0.02 Mandibular posterior teeth: 2.8 ± 0.04
Yanagisawa et al. 2009 (Japan)	547 men (55–75 years) from JPHC Study Cohort I	Self-reported questionnaire administered in 1990, 1995, 2000, and 2005 *Never smokers*: answered “Not smoking” in both 1990 and 2005 and answered “Not smoking” in 1995 and 2000 or had a missing value in 1995 and 2000. *Current smokers*: answered “Currently smoking” in 2005, regardless of answers in 1990, 1995 and 2000. *Former smokers*: answered “Quit smoking” in 2005 or those who answered “Not smoking” in 2005 but “Currently smoking” or “Quit smoking” in 1990.	clinical examination Outcome: having more than 8 missing teeth, mean number of teeth present	% of subjects having more than 8 missing teeth *Never smokers (n* = 161): 28.6% (*n* = 46) *Current smokers (n* = 135): 39.3% (*n* = 53) *Former smokers (n* = 251): 39,0% (*n* = 98) Mean number of teeth present (SE): *Never smokers:* 22.1 (0.6) *Current smokers*: 19.0 (0.7) *Former smokers*: 18.8 (0.5)
Yanagisawa et al. 2010 (Japan)	1088 men (40–75 years of age) resident in Yokote city, Akita prefecture	Self-reported questionnaire *Never smoker:* not defined by the authors *Current smokers:* not defined by the authors *Former smokers:* not defined by the authors The number of cigarettes per day was calculated for current smokers and former smokers, and the smoking-cessation years were calculated for former smokers	clinical examination Outcome: having more than 8 missing teeth, mean number of teeth present	% of subjects having more than 8 missing teeth: *Never smoked (n* = 350): 29.4% (*n* = 87) *Current smokers (n* = 317): 26.2% (*n* = 83) *Former smokers (n* = 421): 26.8% (*n* = 113) Mean number of teeth present (SE): *Never smoked*: 22.0 (0.5) *Current smokers*: 21.4 (0.5) *Former smokers*: 21.4 (0.4)
Yoshida et al. 2001 (Japan)	2015 employees (males, 20–59 years) of a large petroleum chemical plant located in Osaka Prefecture	Self-reported questionnaire *Non-smoking:* not defined by the authors *Quit-smoking:* not defined by the authors *Smoking:* not defined by the authors	Tooth loss: clinical examination	OR (95% CI) for tooth loss *Non-smoking*: 1 (reference) *Quit-smoking*: 1.27 (0.89–1.81) *Smoking: 1.54* (1.20–1.96) * The number of subjects in each group was not informed

SD: standard deviation; CI: confidence interval; OR: odds ratio; SE: standart error

For smoking status assessment, self-administered questionnaires [20, 27, 31, 36–39] and interviews [19, 28, 29, 32–35] were performed. In respect of outcome assessment, tooth loss was determined by clinical examination in ten studies [19, 20, 29, 31–34, 37–39], self-reported questionnaire in three [27, 35, 36] and telephone interview in one [28].

#### Longitudinal studies

Among the 21 included studies, seven [3, 21, 22, 40–43] were longitudinal studies. Their characteristics are shown in Table 2. In total, 70,898 individuals were followed for a period that ranged from 4 to 35 years. Three studies [21, 22, 43] included just males subjects and the other four [3, 40–42] included both males and females.

**Table 2:** Characteristics of the longitudinal studies included in this review (*n* = 07 studies)

Author/ Country/ Follow-up	Follow-up	Subjects	Assessment of smoking status	Assessment of the outcome	Main findings
Dietrich et al. 2007 (EUA)	16 years	43,112 health men professionals with 40 to 75 years from Heatlth Professionals Follow-up Study	Self-reported questionnaire (mailed questionnaire every 2 years) *Never smokers:* < 20 packs of cigarettes in their lives. *Ever smokers*: average number of cigarettes per day. *Former smokers*: years since cessation (time since cessation (< 1, 1–2, 3–5, 6–9, > 10)	Self-reported questionnaire. Subjects reported baseline number of teeth and incident tooth loss in two-year intervals thereafter. Outcome: incident tooth loss	Hazard Ratio (CI 95%) for incidence of first tooth loss: Never: 1.0 (reference) Current (+45cig/d): 3.0 (2.4–3.9) Former (10+ yrs): 1.2 (1.2–1.3)
Dietrich et al. 2015 (Germany)	8.6 years (mean)	21,810 participants from EPIC-Postdam Study with at least 1 natural tooth at baseline	Self-reported questionnaire *Never smokers* *Current smokers:* (< 15 and ≥ 15 cig/day) *Former smokers:* (< 10, 10 to < 20, ≥20 years since cessation)	Self-reported questionnaire. In the last follow-up, patients reported number of natural teeth and the number of teeth lost since study baseline. Outcome: incident tooth loss	Odds ratio (CI 95%) for incidence of tooth loss: *Males <50y* Never: 1.0 (reference) Former smoker ≥20 years: 0.91 (0.66–1.27) Current smokers ≥15 cig/day: 3.64 (3.00–4.42) *Males 50-59y* Never: 1.0 (reference) Former smoker ≥20 years: 1.11 (0.94–1.32) Current smokers ≥15 cig/day: 2.82 (2.36–3.37) *Males 60-70y* Never: 1.0 (reference) Former smoker ≥20 years: 1.18 (0.98–1.44) Current smokers ≥15 cig/day: 2.47 (1.85–3.30) *Females <50y* Never: 1.0 (reference) Former smoker ≥20 years: 0.92 (0.70–1.20) Current smokers ≥15 cig/day: 2.47 (2.11–2.89) *Females 50-59y* Never: 1.0 (reference) Former smoker ≥20 years: 1.20 (0.99–1.44) Current smokers ≥15 cig/day: 2.06 (1.60–2.66) *Females 60-70y* Never: 1.0 (reference) Former smoker ≥20 years: 0.98 (0.78–1.23) Current smokers ≥15 cig/day: 1.79 (1.21–2.63)
Jansson & Lavstedt 2002 (Sweden)	20 years	507 subjects from the population of the Country of Stockholm was perfomerd in 1970 (Lavstedt & Eklund 1975)	Interview Life-time smoking exposure expressed as number of years with a mean consumption of 20 cigarettes per day. *Never smokers*: did not smoke in 1970 and 1990 *Smokers*: smoked in 1970 and 1990 *Former smokers*: stopped smoking between 1970 and 1990	Clinical examination Outcome: Number of teeth lost between 1970 and 1990	Number (SD) of tooth lost: *Never smokers* (*n* = 220): 2.2 (3.0) *Smokers* (*n* = 163): 3.7 (4.8) *Former smokers* (*n* = 124): 3.2 (4.0)
Klein et al. 2004 (EUA)	10 years	2764 subjects (53–96 yrs. of age) from Beaver Dam, WI (1998-2000)	Examiner-administered interview *Never smokers*: persons who smoked 100 or fewer cigarettes in their lifetime. *Current smokers* *Former smokers*	Examiner-administered interview Outcome: Tooth loss (missing some or all teeth)	Odds ratio (CI 95%) for missing some or all teeth: Never: 1.0 (reference) Current: 4.04 (2.52–6.49) Former: 1.57 (1.25–1.98)
Krall et al. 1997 (EUA) (females)*	6 ± 2 yrs. (mean)	584 medically healthy post-menopausal females, screened for nutritional intervention trials at the USDA Human Nutrition Research Center on Aging at Tufts University (Dawson-Hughes et al., 1990).	Annually applied questionnaire *Non-smokers (never or former)*: did not use cigarettes at any time subsequent to baseline. *Continuous smokers:* used cigarettes at baseline and at each subsequent examination. *Quitters*: smoked cigarettes at baseline but reported no cigarette or other tobacco product use at follow-up examinations	Clinical examination and questionnaire. Teeth counted by a nurse practitioner at baseline. Number of teeth lost since baseline and year in which they were lost were assessed by questionnaire at the end of the study. Outcome: Risk of tooth loss; Tooth loss rate/10 yrs	Relative Risk (CI 95%) of tooth loss: *Non-smokers (n* = 225): 1.0 (ref) *Continuous smokers (n* = 09): 3.4 (2.1–5.7) *Quitters (n* = 14): 0.7 (0.3–1.8) Tooth loss rate/10 years *Non-smokers (n = 225):* 0.8 *Continuous smokers (n = 09):* 2.73 *Quitters (n = 14)*: 0.55
Krall et al. 2006 (EUA)	35 yrs. (maximum follow-up)	789 men who participated in the Veterans Administration Dental Longitudinal Study from 1968 to 2004.	Interviewer-administered questionnaire *Never smokers*: men who had never smoked tobacco (cigarettes, pipes, or cigars) either before baseline or during the study *Former smokers*: men who smoked cigarettes before baseline but not during follow-up *Continuous smokers*: men who smoked cigarettes before baseline and continued to smoke cigarettes at each evaluation *Quitters:* men who smoked cigarettes before baseline and quit smoking and abstained from any type of tobacco product	Clinical examination every 3 years Outcome: Tooth loss *per* person, teeth lost *per* year *per* 1000 teeth at risk	No of teeth lost *per* person *Never smokers (n* = 264): 1.0 (0.3) *Former smokers (n* = 283): 1.0 (0.4) *Continuous smokers (n* = 113): 2.0 (0.4) *Quitters (n = 129)*: 3.0 (1.8) No of teeth lost *per* year *per* 100 teeth at risk: *Never smokers (n* = 264): 2 (0.7) *Former smokers (n* = 283): 3 (0.11) *Continuous smokers (n* = 113): 8 (0.17) *Quitters (n* = 129): 7 (2.2)
Okamoto et al. 2006 (Japan)	4 years	1332 (30–59 years) male Japanese	Self-reported questionnaire *Non-smokers*: those did not smoke at either check-up. *Smokers*: smoked at both the baseline and the second check-up. Subdivided into 3 groups based on the number of cigarettes they smoked per day (1–19, 20, or > 21). *Former smokers*: stopped smoking at baseline and had not resumed by the second check-up.	Clinical examination Seven calibrated examiners did the baseline clinical examination and a second examination four years later. Outcome: Teeth lost during 4 yrs	Odds ratio (CI 95%) for tooth loss during 4 years: *Age group 30–39 years:* Never: 1.0 (reference) Former smoker: 0.36 (0.04–3.28) Current smokers 1–20 cig/day: 3.30 (1.09–10.0) Current smokers > 21 cig/day: 2.47 (0.72–8.53) *Age group 40–49 years:* Never: 1.0 (reference) Former smoker: 1.14 (0.59–2.21) Current smokers 1–20 cig/day: 1.48 (0.76–2.91) Current smokers > 21 cig/day: 2.03 (1.00–4.10) *Age group 50–59 years:* Never: 1.0 (reference) Former smoker: 1.07 (0.44–2.61) Current smokers 1–20 cig/day: 1.34 (0.49–3.68) Current smokers > 21 cig/day: 1.67 (0.56–4.99)

cig/d: cigarette *per* day; SD: Standard Deviation

* just the women group was considered. Men group was analyzed in the Krall et al. 2006 study

Smoking behavior was determined using self-reported questionnaires [22, 40, 42, 43] or interviews [3, 21, 41], whereas tooth loss was assessed by clinical examination [3, 21, 43], self-reported questionnaires [22, 40, 42] or interviews [41].

### Methodological quality of included studies

#### Cross-sectional studies

Risk of bias assessment of the cross-sectional studies was evaluated according to the NOS domains (Table 3). Of the 14 cross-sectional studies included, five (35.7%) were considered to have low risk of bias [28, 29, 33–35], seven (50%) presented moderate risk [19, 20, 27, 31, 36–38] and two (14.3%) [32, 39] were judged to have high risk of bias.

**Table 3:** Risk of bias assessment of included cross-sectional studies

	Selection (maximum 5)	Comparability (maximum 2)	Outcome (maximum 3)	Total (maximum 10)
Albandar et al. 2010	1 ★	1 ★	3 ★	5 ★
Arora et al. 2010	3★	1 ★	1 ★	5★
Cunningham et al. 2016	4 ★	2 ★	1 ★	7 ★
Hanioka et al. 2007	3 ★	1 ★	3 ★	7 ★
Mai et al. 2013	1 ★	2 ★	3 ★	6★
Mundt et al. 2007	3 ★	1 ★	3 ★	3 ★
Musacchio et al. 2007	3 ★	1 ★	3 ★	7 ★
Ojima et al. 2007	3 ★	1 ★	3 ★	7 ★
Randolph et al. 2001	4 ★	2 ★	1 ★	7 ★
Simila et al. 2006	2 ★	2 ★	1 ★	5 ★
Torrungruang et al. 2012	0 ★	2 ★	3 ★	5 ★
Yanagisawa et al. 2009	1 ★	1 ★	3 ★	5 ★
Yanagisawa et al. 2010	2 ★	1 ★	3 ★	6★
Yoshida et al. 2001	0 ★	1 ★	3 ★	4 ★

Scores ranged from 0 to 10 stars. Studies with 7–10 stars were arbitrarily rated as low risk of bias, 5–6 stars moderate risk of bias and < 5 high risk of bias

#### Longitudinal studies

Risk of bias of the longitudinal studies is shown in Table 4. None of the included studies were considered to have high risk of bias. Most of the studies [21, 22, 40–43] were considered to have moderate risk of bias and just one study [3] presented a low risk of bias.

**Table 4:** Methodological quality of the longitudinal studies

	Selection (maximum 4)	Comparability (maximum 2)	Outcome (maximum 3)	Statistics (maximum 2)	Total (maximum 11)
Dietrich et al. 2007	2 ★	2 ★	2 ★	2 ★	8★
Dietrich et al. 2015	2 ★	2 ★	2 ★	2 ★	8 ★
Jansson & Laystedt. 2002	4 ★	1 ★	2 ★	2 ★	9 ★
Klein et al. 2004	3 ★	1 ★	1 ★	1 ★	6 ★
Krall et al. 1997	3 ★	0 ★	1 ★	2 ★	6 ★
Krall et al. 2006	3 ★	1 ★	2 ★	2 ★	8 ★
Okamoto et al. 2006	1 ★	1 ★	2 ★	2 ★	6 ★

Scores ranged from 0 to 11 stars. Studies with 9–11 stars were arbitrarily rated as low risk of bias, 6–8 stars moderate risk of bias and < 6 high risk of bias

### Pooled outcomes

#### Cross-sectional studies

**Fig. 2:**
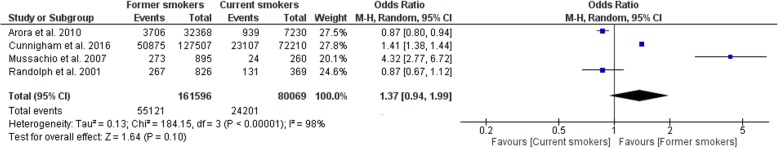
Forest plot for meta-analysis of being edentulous in former smokers compared to never-smokers in cross-sectional studies (*n* = 04 studies, association measure: Odds ratio)

**Fig. 3:**
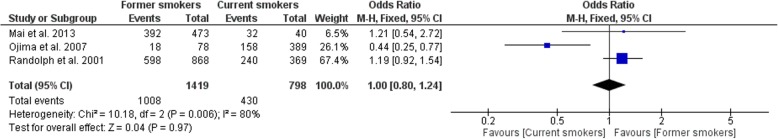
Forest plot for meta-analysis of losing 1 or more teeth in former smokers compared to never-smokers in cross-sectional studies (*n* = 03 studies, association measure: Odds ratio)

**Fig. 4:**
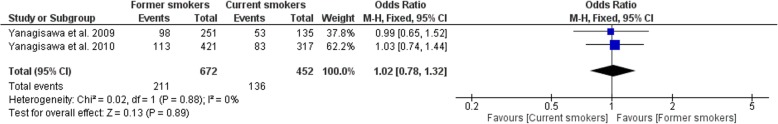
Forest plot for meta-analysis of losing more than 8 teeth in former smokers compared to never-smokers in cross-sectional studies (*n* = 02 studies, association measure: Odds ratio)

Concerning cross-sectional studies, a total of three meta-analyses comparing former vs current smokers were conducted. Former smokers were compared to current smokers as regards number of: (i) edentulous subjects, (ii) patients who lost 1 or more teeth and (iii) patients who lost more than 8 teeth. Pooled estimates comparing former vs current smokers showed no significant difference in the odds of being edentulous (OR = 1.37; 95% CI = 0.94 to 1.99, Heterogeneity: I^2^ = 98%, *p* = 0.10), losing 1 or more teeth (OR = 1.00; 95% CI = 0.80 to 1.24, Heterogeneity: I^2^ = 80%, *p* = 0.97) and losing more than 8 teeth (OR = 1.02; 95% CI = 0.78 to 1.32, Heterogeneity: I^2^ = 0%, *p* = 0.89). (Figs. 2, 3 and 4).

#### Longitudinal studies

**Fig. 5:**
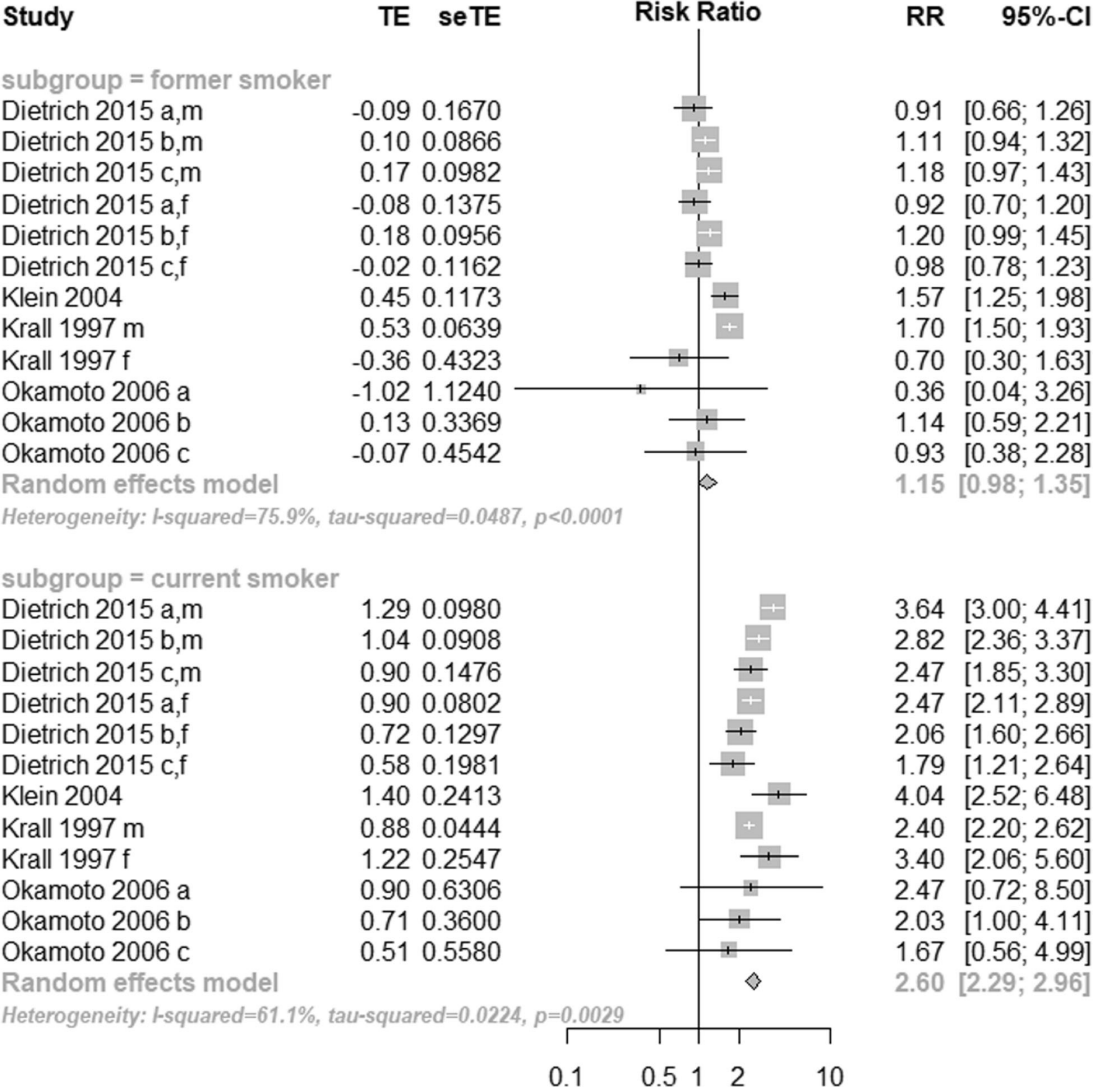
Forest plot for meta-analysis of tooth loss in current and former smokers compared to never-smokers in longitudinal studies (*n* = 04 studies, association measure: Risk Ratio). M: male, f: female. In the Dietrich 2015 study, **a**: < 50 years old, **b**: 50–59 years old, **c**: 60–79 years old. In the Okamoto 2006 study, a: 30–39 years old, b: 40–49 years old, c: 50–59 years old

The risk of tooth loss among those who quit smoking was not significantly different from never smokers. Contrarily, current smokers presented a risk of tooth loss twice higher than never smokers. Moderate to high level of heterogeneity was found even within the subgroups (Fig. 5).

Among former smokers, the time of cessation was the variable that explained a considerable part of the heterogeneity (around 60%), resulting in a low residual heterogeneity (around 20%) when included in the regression model (Table 5). However, the level of significance was not inferior to 5%. On the other hand, for current smokers, no moderator was significantly associated with the estimates (Table 5). The funnel plots showed no visual or statistically tested asymmetries (Additional file 1 a and b).

**Table 5:** Meta-regression analysis for the association between moderators and tooth loss

	Former Smokers				Current smokers			
Moderator	Estimate* (95%CI)	*p*-value	Residual I2	R2	Estimate* (95%CI)	p-value	Residual I2	R2
Age	0.01 (−0.87 to 0.38)	0.30	0%	0%	−0.01 (−0.03 to 0.005)	0.15	77.6%	0%
Cigarretes	−0.01 (− 0.10 to 0.07)	0.76	0%	0%	− 0.04 (− 0.14 to 0.06)	0.43	64.7%	0%
Dropout rateƗ	0.01 (−0.01 to 0.02)	0.50	74.1%	0%	0.004 (−0.01 to 0.02)	0.58	70.2%	43.7%
Time since cessation	0.09 (−0.01 to 0.18)	0.06	22.0%	57.8%	–	–	–	–

CI: Confidence Interval, I2: residual heterogeneity / unaccounted variability, R2: amount of heterogeneity accounted for each variable, * Coefficient of Linear Regression (meta-regression), Ɨ dropout rates considering the entire sample included in the study (or the any subgroup, when available)

## Discussion

The aim of this systematic review was to assess if smoking cessation reduces the risk of tooth loss in former smokers, when compared to current smokers. Our results showed that smoking cessation may reduce the risk of tooth loss. Meta-analysis of data from longitudinal studies showed that the rate of tooth loss in former smokers is similar to that of never smokers. Moreover, current smokers had a risk of tooth loss twice higher than never smokers. These results are consistent with a previous systematic review that found a causal relationship between smoking and tooth loss and a decreased risk of tooth loss in former smokers [17]. However, the effect of smoking cessation on tooth loss had not been explored in this previous review. To the best of the authors’ knowledge, this is the first systematic review with meta-analysis that included never, former and current smokers, as different levels of exposures to smoking and investigated their association to tooth loss.

The most plausible biological explanation for the increased risk of tooth loss in smokers is the destruction of the periodontal supporting tissues [17]. A recent systematic review showed that the risk for periodontitis incidence and progression could be reversed after smoking cessation to the same level as that of never smokers [12]. These results are in agreement with our findings that the risk of tooth loss between former smokers and never smokers were not significantly different.

In contrast to the results from longitudinal studies, the meta-analysis of data from cross-sectional studies did not show significant differences between former smokers and current smokers in relation to the risk of being edentulous, losing one or more teeth and losing eight or more teeth. The possible reason for this lack of effect is the inherent limitations of cross-sectional studies, especially the absence of information about the temporal relationship between cause (smoking cessation) and effect (tooth loss). For example, none of the included cross-sectional studies assessed the time of tooth loss. Thus, it is possible that former smokers lost their teeth before stopped smoking. Moreover, most of these studies failed to report the non-smoking duration for subjects that ceased the habit. It is possible that many quitters in the included studies have stopped smoking for less than 5 years. Considering that it may take at least 10 to 20 years of abstinence for the risk to return to the level of never smokers [21, 22, 40], the inclusion of recent quitters in the analysis could have reduced the effect size of smoking cessation on tooth loss in the cross-sectional studies.

Even though our meta-estimates were derived from observational studies, which usually present high heterogeneity, the results provided from the analysis of this kind of studies can be considered similar to those of randomized trials [50]. Besides, heterogeneity was considered in our meta-analyses (random-effects models) and explored (subgroup and meta-regression analyses), contributing to an appropriate judgment about the findings and helping in identifying potential sources of heterogeneity. In addition, another point that should be emphasized is that the sample size in observational studies is frequently larger than that of clinical trials. Altogether, the studies included in this review enrolled 638,389 individuals (567,491 subjects from cross-sectional studies and 70,898 participants from longitudinal studies). Moreover, there are methodological difficulties in using tooth loss as an outcome in interventional studies. It would be necessary a very long follow-up time and a very large sample size to analyze this outcome.

Some methodological differences between the studies should be pointed out. For example, while some studies [22, 40] asked the time since smoking cessation at the baseline questionnaire, others [3, 21, 41–43] considered as former smokers the subjects that stopped smoking during the follow-up time. These methodological differences could have influenced the results since in the first case [22, 40] participants could have stopped smoking many years before tooth loss. Time since cessation was the variable that better explained the heterogeneity in meta-regression. However, the effect was not significant, which could be related to the small number of studies included in this analysis. Another important consideration that has to be pointed out is that all included studies were carried out in high-income countries. It is necessary to be carefully to extrapolate our results to low-middle-income countries because it is known that socio-economic differences have an important role in oral health status, tooth loss and smoking status. Well-conducted studies with these populations are necessary.

None of the studies have used an objective measure of smoking status (e.g. salivary levels of cotinine or levels of carbon monoxide exhaled). Self-reported smoking status has been associated with underestimated smoking prevalence [51]. Along with the same lines, self reported tooth loss may not be accurate. Although clinical examination is the best method to determinate tooth loss, some studies [27,28,53,36,22,40–42] used self-report to determinate this outcome. This method could have been chosen because of the high number of participants or the long follow-up time (longitudinal studies). Another shortcoming in the included studies was that the reason of tooth loss was not considered. This information could help to better explain the relation between smoking and tooth loss.

Despite the methodological limitations of the included studies, the findings of this systematic review support a beneficial effect of smoking cessation on the risk of tooth loss. Considering the benefits of quitting tobacco for the general health, and that smoking cessation interventions conducted by oral health professionals are effective [52], the dental setting seems to be appropriate to implement smoking cessation therapy.

## Conclusions

This systematic review indicates that risk for tooth loss in former smokers is comparable to that of never smokers. Moreover, current smokers present a higher risk of tooth loss than former smokers.

## Supplementary information

**Additional file 1. **Funnel plots of longitudinal studies investigating the effect of smoking and smoking cessation on tooth loss. (a) former smokers vs. never smokers (Egger’s test for asymmetry, *p* = 0.06), (b) current smokers vs. never smokers (Egger’s test for asymmetry, *p* = 0.79).

## Data Availability

All data generated or analyzed during this study are included within the article (and its additional files).

## Abbreviations

CI: confidence interval
MOOSE: Meta-analysis of Observational Studies in Epidemiology
NOS: Newcastle-Ottawa scale
OR: odds ratio
PRISMA: Preferred reporting items for systematic reviews and meta-analyses
PROSPERO: International Prospective Register of Systematic Reviews
RR: risk ratio
