# Supplementary figures and images for: Effect of smoking cessation on tooth loss: a systematic review with meta-analysis
Source: BMC Oral Health. 2019 Nov 12;19:245. doi: 10.1186/s12903-019-0930-2 (PMC6852780; doi:10.1186/s12903-019-0930-2)

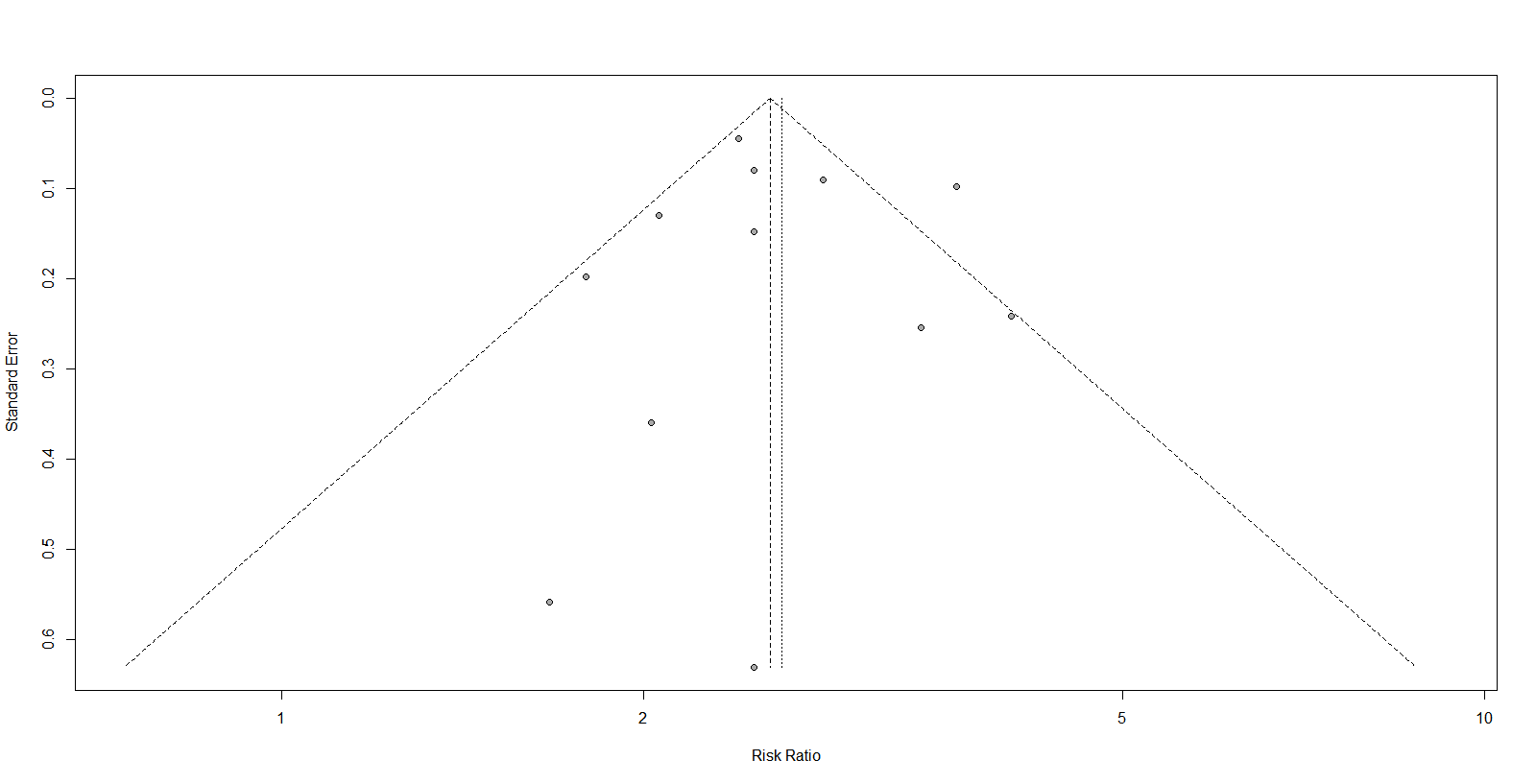

Supplement: Supplementary file 1 — Additional file 1. Funnel plots of longitudinal studies investigating the effect of smoking and smoking cessation on tooth loss. (a) former smokers vs. never smokers (Egger’s test for asymmetry, p = 0.06), (b) current smokers vs. never smokers (Egger’s test for asymmetry, p = 0.79). [file 12903_2019_930_MOESM1_ESM.zip › Supplemental file 1b funnel currentR3.tif]

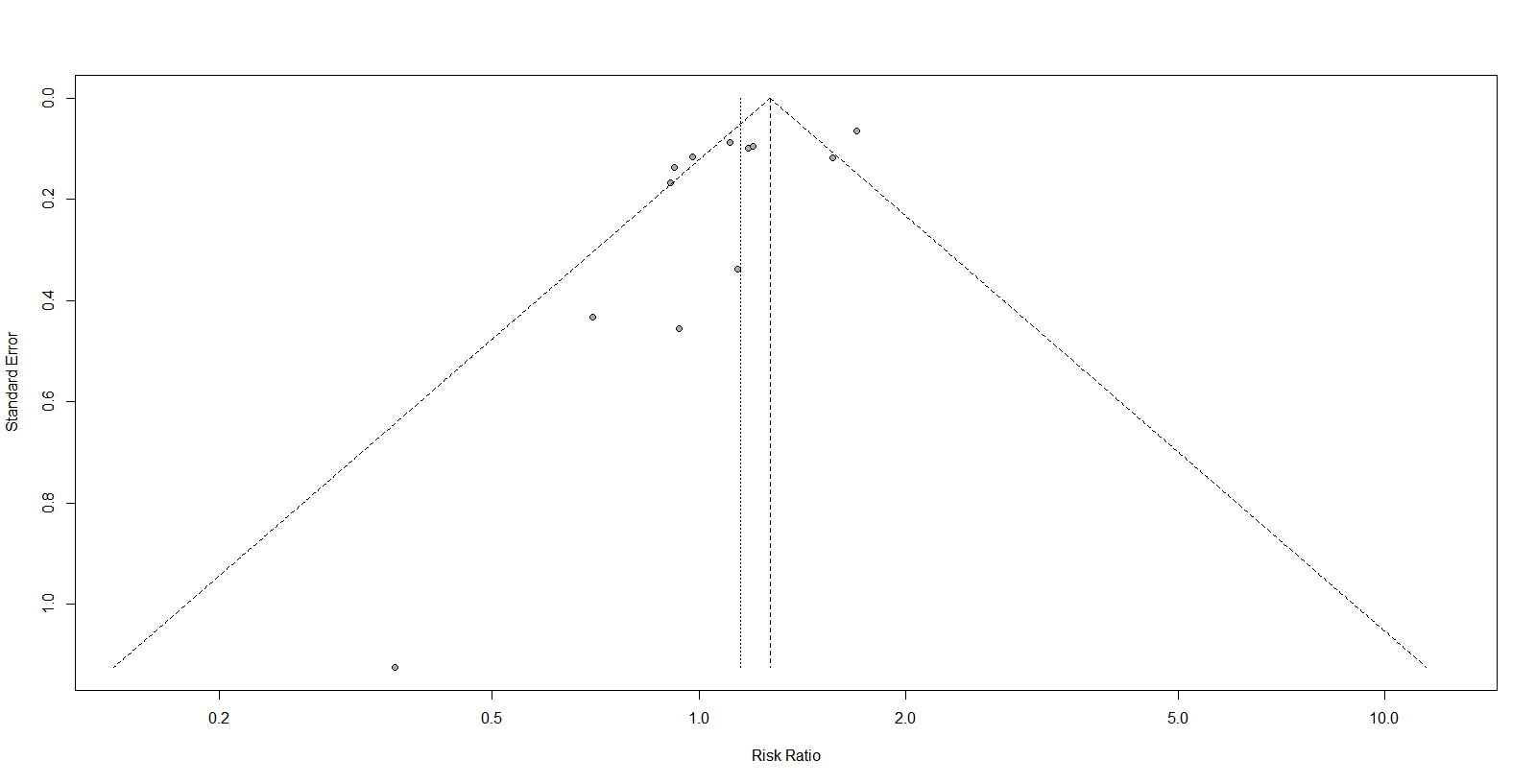

Supplement: Supplementary file 1 — Additional file 1. Funnel plots of longitudinal studies investigating the effect of smoking and smoking cessation on tooth loss. (a) former smokers vs. never smokers (Egger’s test for asymmetry, p = 0.06), (b) current smokers vs. never smokers (Egger’s test for asymmetry, p = 0.79). [file 12903_2019_930_MOESM1_ESM.zip › Supplmental file 1a funnel formerR3.tif]
